# Supplementary material for: Are carbon emissions trading and green financial instruments synergistic? -Comprehensive quantitative research based on content analysis
Source: PLoS One. 2024 Mar 7;19(3):e0298601. doi: 10.1371/journal.pone.0298601 (PMC10919601; doi:10.1371/journal.pone.0298601)
Supplement: S1 Data — (ZIP) [file pone.0298601.s001.zip › Data/地级市Z_F工作报告.zip!/║╙▒▒/└╚╖╗╩╨/└┤╫╘╣1⁄2╓┌║┼í╛┬φ┐╦╩2╛▌═°í┐_2014.docx]

各位代表：

　　现在，我代表廊坊市人民政府向大会作工作报告，请予审议，并请市政协委员和列席会议的同志提出意见。

　　一、2013年主要工作回顾

　　刚刚过去的2013年，面对严峻复杂的经济形势，我们在省委、省政府和市委的坚强领导下，在市人大、市政协的监督支持下，深入贯彻落实党的十八大和十八届三中全会精神，以习近平总书记系列重要讲话为指导，坚持解放思想、改革开放、创新驱动、科学发展，奋力打造环京津新增长极战略突破口，较好地完成了市六届人大一次会议确定的目标任务。

　　经济发展稳中有进。针对经济下行压力加大、环境治理任务艰巨的困难形势，多措并举保运行，千方百计稳增长，经济保持平稳发展态势。全市生产总值完成1943.1亿元，同比增长9.1%；全部财政收入349.2亿元、公共财政预算收入205.4亿元，分别增长13.9%和19.3%；全社会固定资产投资1577.8亿元，增长20.1%；规模以上工业增加值706.6亿元，增长10%；社会消费品零售总额637.4亿元，增长13.4%。经济发展质量效益明显提升，全部财政收入占生产总值比重达到18%，公共财政预算收入占全部财政收入比重达到58.8%，分别提高0.9和2.7个百分点；公共财政预算收入中税收占比达到87.1%，提高2.4个百分点。

　　项目建设力度加大。坚持把项目作为经济发展的核心抓手，集中攻坚，持续加力，取得显着成效。全市建设亿元以上项目882项，同比增加37项，其中10亿元以上项目138项，同比增加19项。争列省管重点项目14项、市管省考核项目48项，均居全省前列，项目开工率、年度投资完成率实现或超过100%，为历年最高。润泽国际信息港一期、美国JM新型管材、中建机械二期、香江全球家居CBD、达利食品等一批重大项目竣工投产，航天科技产业园、联通华北基地等一批项目加快推进，发展后劲持续增强。

　　产业结构不断优化。坚持“无中生有、有中生新”，实施创新驱动战略，产业结构调整取得新进展。新兴产业不断壮大。电子信息产业主营业务收入实现253亿元，产业规模、增速保持全省领先，廊坊开发区成为国家级电子信息产业基地。高端装备制造、生物医药、新材料、新能源等产业加快聚集，中轻造纸装备二期、新奥光伏示范基地等一批重大项目开工建设。金融、物流、研发等现代服务业强势发展，服务业增加值增长12.8%。现代农业蓬勃发展，农业产业化经营率达到66.5%，居全省领先。传统产业加快转型。设立5000万元工业技改专项资金，实施技改项目570项，新增规模以上工业企业163家，国家级金属玻璃家具质检中心落户胜芳。科技创新成效明显。新增高新技术企业12家，累计达到252家，高新技术产业增加值增长14.4%。争列国家级科技项目43项，5项列入国家863计划。国家可持续发展实验区主体工程通过验收，龙河园区被批准为省级高新技术产业开发区，大厂农业园区成为省级现代农业科技示范园区，我市连续第六次被评为全国科技进步先进市。

　　重点区域发展步伐加快。全力推动重点区域、园区率先突破，努力打造环京津新增长极战略突破口的引爆点。临空经济区启动建设。首都新机场建设前期工作加快推进，机场布局、征迁补偿、交通体系对接等更多体现廊坊利益诉求。临空经济区建设上升为国家战略，京冀共同编制总体规划，我市区域概念规划和产业规划基本完成。北三县统筹发展破题开局。围绕打造京津冀协同发展合作示范区、绿色发展先行区和城乡统筹试验区，组建专门推进机构，积极推进北三县统筹发展的产业和空间规划编制工作，基础设施共建、产业布局优化、生态环境建设等全面展开。园区建设得到加强。实施精品园区发展战略，制定精品园区评价办法和指标体系，促进园区上档升级。推进区中园建设，谋划建设电梯产业园、管道产业园、环保产业园等专业园区。省级以上园区利用外资、固定资产投资分别占到全市的88%和78.6%，园区承载力、带动力进一步提升。

　　城乡建设水平显着提高。坚持城乡统筹，加快新型城镇化步伐，城乡面貌发生新变化。城镇建设扎实推进。城市总规修编基本完成，县级城乡总规和近期建设规划修编全面展开。重大城建工程顺利推进，生态文化艺术新区成为中美低碳生态城市试点，高铁站南广场投入使用，金光道地下通道等重点工程加快建设，艺术大道东延等6条主次干道建设全部完工。开展城市环境整治百日攻坚，实施“净化、绿化、亮化、美化、序化”五大工程，城市精细化管理得到加强。全面实施县城建设“五个一”工程，县城综合承载力明显增强。重大基础设施建设全面提速。京台高速、密涿高速加快建设，南水北调廊涿干渠、天津干渠具备通水条件，保沧干渠建设顺利实施，8个地表水厂启动建设，国电廊坊热电厂项目开工建设，中电投热电项目加快前期工作。农村面貌持续改善。深化基层建设年活动，实施农村面貌改造提升行动，村庄“四清”任务基本完成，133个省级重点村全面落实道路硬化、饮水安全等15件实事，新建改建农村公路150公里，农村生产生活条件进一步改善。

　　环境治理力度空前。围绕改善空气质量，铁腕治理大气污染，全面打响燃煤、扬尘、尾气、烟气和工业污染五大治理攻坚战。全市拆除燃煤锅炉437台、脱硫除尘改造102台，钢铁企业全部实现脱硫除尘改造和在线监测。实施城中村散烧煤专项治理，建筑工地强力落实“六个100%”，城市环卫基本实现湿扫。全市淘汰黄标车62729辆，超额完成省下达任务。关停取缔拆除重污染企业367家，三河拆除全部169座马蹄窑、关停所有采石企业，文安关停整改胶合板、钢铁、塑料企业520家，大城停产整改岩棉企业150家，霸州、永清彻底取缔非法小电镀企业。强化环境执法和监测，市县两级成立公安环保支队，开展“利剑斩污”专项行动，在全省率先完成县级空气质量监测站建设，文安率先建立网格化环境监管体系。全面完成淘汰落后产能任务，压减炼铁88万吨、平板玻璃70万标准重量箱。超额完成省下达节能减排任务，实施188项减排工程，新建8个污水处理厂，尹家沟、鲍邱河水污染整治取得阶段性成果。大规模开展植树造林，全市造林20.2万亩，市区新增绿化面积57.6万平方米。市区大气污染治理取得阶段性成效，下半年PM2.5日均值浓度比上半年下降19.7%，达标天数比上半年增加28天。

　　改革开放有序推进。把改革开放作为破解难题、加快发展的根本动力，进一步释放发展活力。各项改革取得新进展。大力简政放权，取消下放市级行政审批事项65项。财政预算形成链条化审核机制，在全国地级市率先推行国库集中支付电子化管理，顺利推进“营改增”改革。事业单位改革基本完成岗位设置方案审批，5家国企完成改制，5家企业在天交所和石交所挂牌上市。医药卫生体制改革取得新进展，基层医疗卫生服务体系基本实现城乡全覆盖，38家二级以上医院、所有乡镇卫生院和67%的政府办村卫生室实行基本药物制度，8家县级医院开展公立医院改革试点。农村产权制度改革深入推进，农村土地承包经营权流转比例达到14.9%。对接京津进入新阶段。抓住京津冀协同发展新契机，更加积极地承接京津产业外溢和功能疏解，开展赴京津系列招商活动，在首都教育、科技、医疗、会展、市场转移和交通对接等方面，达成一批合作协议。招商引资成效显着。廊坊经洽会、“9·26”农交会圆满成功，签约项目位居全省前列，举办各类专业展会49个。组织开展小团组招商、点对点招商、产业链招商和园区招商等专题招商活动，实际利用外资6.85亿美元，增长10.5%。金融支撑得到增强。浦发银行、张家口商业银行入驻我市，市级银行业金融机构达到15家。农村信用社改制、村镇银行组建加快推进，金融网点实现乡镇全覆盖。加强对小额贷款及担保机构的监管，金融组织体系进一步完善。

　　民生社会事业全面发展。全年财政用于民生支出224.1亿元，占公共财政预算支出的77.9%。城镇居民人均可支配收入26985元，农民人均纯收入11624元，分居全省第一、第二位。新增就业5.64万人，城镇登记失业率保持全省最低。市第三人民医院正式接诊，廊坊一中迁建和市中心医院建设完成立项、设计、拆迁等前期工作，具备开工条件。市城区新改扩建8所中小学和幼儿园，全市创建152所标准化学校。投资30.8亿元，完成576所学校改造，校安工程圆满收官。城乡低保应保尽保，五保集中供养率全省第一，企业退休养老金增长12.3%，职工住院费起付标准大幅降低，居民门诊大病报销范围和支付限额进一步扩大。开工建设保障性住房1.71万套、竣工1.33万套，8648户群众喜迁新居。改造提升32个无物业老旧小区，8000多户居民直接受益。住房公积金累计归集111.3亿元、发放贷款55.5亿元。投资1.36亿元，完成22座农村集中供水水厂主体工程，农村饮水安全问题基本解决。举办“百名画家画廊坊、百名摄影家拍廊坊”、京津廊戏曲大奖赛等系列群众性文化活动，全民健身活动蓬勃开展。平安建设深入实施，开展“京畿亮剑”和打击“两抢一盗”专项行动，安全生产、食品药品安全、信访稳定工作进一步加强，我市被中央综治委评为全国社会管理综合治理优秀市，香河荣获全国综治“长安杯”。国防后备力量建设得到加强，双拥共建深入开展，人口计生、民族宗教、广播电视、妇女儿童、防灾减灾、外事侨务、气象、档案、人防、旅游、残疾人等各项事业取得新成绩，涌现出全国公安二级英模陈彩凤、霸州“侯振国爱心团队”等一批先进典型，和谐稳定局面得到巩固，社会正能量进一步凝聚。

　　政府自身建设不断加强。积极转变政府职能，扎实推进作风转变，政府效能和行政能力进一步提高。严格落实“八项规定”，开展正风肃纪专项行动，全面清理处置“吃空饷”人员、违规公务用车和超标办公用房，市级“三公”经费下降6%，“四风”问题得到遏制。深入推进效能廊坊和廉洁廊坊建设，完善规范性文件和重大决策合法性审查机制，严厉整治不作为、乱作为问题，行政权力制度化、规范化、法治化程度得到提升。畅通群众诉求渠道，坚持重要事项公示和听证制度，市长专线受理答复群众来电53625件，及时解决了一大批群众反映的困难和问题。自觉接受人大法律监督、工作监督和政协民主监督，办理人大代表建议149件、政协提案376件，办复率均为100%。

　　各位代表！面对宏观经济形势严峻复杂的局面，特别是面对骤然增加的大气污染治理和传统产业转型升级带来的压力，我市经济社会发展取得的成绩来之不易，弥足珍贵。这是省委、省政府和市委科学决策、正确领导的结果，是市人大、市政协有效监督、大力支持的结果，是全市人民拼搏进取、团结奋斗的结果。在此，我代表市人民政府，向全市人民，向人大代表、政协委员，向各民主党派、人民团体和各界人士，向驻廊解放军、武警官兵、政法干警和中省直驻廊单位，向所有关心支持廊坊发展的朋友们，致以崇高的敬意和衷心的感谢！

　　我们也清醒地认识到，我市经济社会发展和政府工作还存在不少差距和问题。尽管去年大多数目标任务完成或超额完成，但仍有规模以上工业增加值、社会消费品零售总额等指标没有达到预期目标。经济发展中的深层次问题尚未根本解决，传统产业档次低转型慢、新兴产业规模小实力弱的问题日益凸显，园区档次、城镇功能、城乡基础设施和公共服务能力亟待提高，土地、资金等要素制约依然明显，大气和水污染以及安全生产等事关群众切身利益的问题解决得还不够好，淘汰过剩产能、节能减排和生态环境建设任务十分艰巨。一些部门和工作人员服务意识不强、办事效率不高，干部队伍中小进即满意识滋长，消极腐败现象还不同程度存在。我们一定正视问题，本着对党和人民高度负责的精神，采取更加有力的措施认真加以解决，决不辜负全市人民的期望！

　　二、2014年工作总体要求

　　今年是贯彻落实党的十八届三中全会和省委八届六次全会精神、全面深化改革的第一年，是落实市委五届六次全会精神、实现“十二五”目标任务的关键之年。纵观今年面临的形势，发展压力超越以往，从宏观环境看，经济发展的不确定因素依然较多，我省已进入经济增长换挡期、结构调整阵痛期和前期刺激政策消化期相叠加的特殊阶段。从我市来说，产业结构调整和环境保护给我们带来的压力前所未有，经济运行中的矛盾和困难更为突出。发展要求超越以往，省委、省政府对廊坊发展寄予厚望，明确要求我们要打造好临空经济区和北三县统筹发展两大引爆点，建设好拱卫首都环境护城河，在推进新型城镇化方面做示范，在京津冀协同发展中当先锋，自觉担当起打造河北环京津新增长极战略突破口的重任。发展机遇超越以往，京津冀协同发展和环渤海地区加速崛起，更加凸显了我市在区域发展中的重要地位，为廊坊更好融入京津、借势京津提供了有利条件。同时，首都经济圈规划加快编制并上升为国家战略，首都新机场和临空经济区启动建设，首都产业、功能加快向外转移，都为廊坊发展创造了重大机遇。我们必须把压力变为动力，把期望化为担当，进一步增强机遇意识、进取意识、责任意识，推动廊坊经济社会发展迈上新台阶。

　　今年政府工作的总体思路是：以党的十八大、十八届三中全会、省委八届六次全会和市委五届六次全会精神为指导，切实转变经济发展的指导思想，切实推进体制机制改革创新，牢牢把握环渤海加速发展和京津冀协同发展的重大机遇，牢牢把握“绿色、高端、率先、和谐”的发展定位，更加突出保护生态环境，更加突出发展实体经济，更加突出改善民生，勇于担当，强势推进，狠抓落实，加快打造河北环京津新增长极战略突破口。

　　今年经济社会发展的主要预期目标是：全市生产总值增长8.5%左右，公共财政预算收入增长10.5%，全社会固定资产投资增长17%，社会消费品零售总额增长13%，实际利用外资增长5%，城镇居民人均可支配收入、农民人均纯收入分别增长8.5%以上和9%左右，节能减排指标完成省下达的目标任务，PM2.5浓度下降5%以上。提出这样的指标，综合考虑了我市发展的需要与可能，并与“十二五”规划、全面建成小康社会目标相衔接，既不盲目追求高增长，也避免因为“换挡”造成“失速”，更加突出经济增长的质量和效益，更加注重环境改善和可持续发展，为全面深化改革、加快结构调整和生态环境治理创造条件、留出空间。

　　实现今年经济社会发展目标，必须坚持“稳中求进、改革创新”的工作总基调，以建设平原森林城市、环保产业城市、文化体验城市、幸福宜居城市为目标，以全力实施十项集中行动为重点，着力在“重改革、调结构、优生态、强支撑、统城乡、惠民生”上下功夫。重改革就是把改革创新贯穿于经济社会发展各领域各环节，以改革促创新、促发展、促转型。调结构就是坚持“无中生有、有中生新”，突出发展实体经济和新兴产业，扎实推进传统产业转型升级，积极打造环保型产业体系。优生态就是坚定不移走绿色崛起之路，全面治理环境污染，更加注重生态建设，使绿色成为廊坊最鲜明的城市特征，重塑廊坊环境优势。强支撑就是准确把握对接京津的共振点、结合点，加快形成新的引爆点、突破点，在重点区域和园区建设上实现新突破。统城乡就是立足廊坊特殊区位和有利条件，加大城乡协同发展力度，按照省委“十要十不要”的要求，努力走出一条以人为本、优化布局、生态文明、传承文化的新型城镇化道路。惠民生就是切实解决好群众最关心、最迫切、最现实的问题，严守民生底线，多办实事好事，确保人民安居乐业、社会和谐稳定。

　　三、坚定不移全面深化改革

　　坚持以改革统领全局，围绕使市场在资源配置中起决定性作用和更好发挥政府作用，正确准确有序协调推进各项改革，以改革增动力、强活力。

　　（一）深化行政管理体制改革。加快转变政府职能，切实把政府职能转到为各类市场主体创造统一开放、竞争有序的市场环境上来。一是进一步简政放权。认真落实省取消和下放的行政审批事项，全面清理市本级行政审批事项，应取消的坚决取消，应下放和能下放的坚决下放到位。积极配合做好省向县、县向乡镇直接放权的试点工作。二是推进行政审批体制机制改革。加强市县“一办两中心”建设，强化人员力量，完善机制流程，提高服务水平。推进行政审批标准化，制定并公开行政审批事项目录，没有进入目录的一律停止审批。加强电子政务网络平台建设，提高网上审批服务效率，让群众少跑路、少受累。加强行政监管方式创新，推进政府管理由事前审批向事中、事后监管转变。三是进一步提高政府效能。全面完成市县政府机构改革，扎实开展政府机关标准化建设，积极稳妥推进市区城市管理体制改革，继续实施执法监管部门和窗口单位提质提效专项行动，实行“零障碍”服务全程协办机制。全面整合各类民生服务热线电话，更好受理群众诉求。

　　（二）深化重点领域改革。一是大力推进财税金融改革。加快预算管理制度改革，完善政府预算体系，建立跨年度预算平衡机制，规范预决算公开内容和程序，推进“营改增”试点扩围。强化政府债务管理，建立财政风险预警机制，规范新增债务审批程序和风险等级控制，有序化解债务风险。加强域内外金融合作，搭建政金企对接平台，推进农村信用社改制和村镇银行发展。强化部门监管职责，加大对小额贷款公司、担保机构及各类投融资中介机构监管力度，严密防范金融风险。制定专项支持政策，鼓励更多企业上市，努力实现县县有上市企业。二是深入推进国企改革。完善国有资产监管体制，大力推进国企改制重组，鼓励国有资本与其它性质资本交叉持股、融合发展，大力发展混合所有制经济，确保国有资产保值增值。三是稳妥推进农村综合改革。依法保障农民对承包地占有、使用、收益、流转及承包经营权抵押、担保权利，建立健全土地承包经营权流转市场，加快农村土地确权登记颁证，促进土地承包经营权流转和规模集约经营。引导工商资本发展现代农业，支持农民开展专业合作、股份合作，扶持现代农业示范园区、家庭农场、农民合作社等新型农业经营主体，加快构建新型农业经营体系。四是积极推进社会事业改革。统筹城乡教育资源均衡配置，完善贫困生资助体系，鼓励社会力量兴办教育。深入推进县级公立医院和基层医疗机构综合改革，积极引导社会资本办医，有序扩大基本药物制度实施范围。启动实施“单独两孩”政策。稳妥推进市城区水务一体化改革，加快实现水资源统一管理。五是努力推进基层管理改革。城区重点推进社区信息化平台和社工队伍建设，实施政府购买社会服务试点。农村积极推行“四个全覆盖”，构建基层综合服务管理平台，不断提升基层服务管理水平。

　　（三）促进民营经济创新发展。坚持民营与国有平等发展的导向，不断优化民营经济发展环境。一是推进投资体制改革。实行非禁即准、非限即许，放开民间投资领域，鼓励民营经济参与国企改革，进入特许经营领域和控股混合所有制企业。二是推进商事登记制度改革。由先证后照改为先照后证，实行投资主体零限制、注册零收费，把注册资本实缴登记制改为认缴登记制，放宽市场主体注册资本、住所登记条件，企业年检制度改为年报制度，大力度推进全民创业，大幅度增加市场主体。三是提升服务水平。开展服务民企集中行动，实施促进民企成长工程，选择一批成长性好、发展潜力大的民营企业，由市县两级领导干部重点联系，积极宣传、全面落实鼓励民营经济发展的各项政策措施，在土地、资金等要素资源上加大支持力度，打造“民企航母”。强化中小企业公共服务平台、融资平台和创业辅导基地功能，加大企业家培训力度，促进“个转企、小升规”。力争全年新增规上企业180家，新增注册民营企业2000家。

　　（四）深化对接京津。找准合作结合点、共振点，推进规划衔接、产业对接、功能承接、交通联接，在京津冀协同发展中抢占先机。围绕北京功能疏解及要素外溢的需求，大力宣传廊坊的城市、产业、园区、环境，提升廊坊的竞争力和吸引力。顺应北京向外转移社会资源的趋势，加大对科技、教育、医疗、会展、专业市场等功能性资源的承接力度，积极吸引京津人才资源，推进北京优质医疗资源、重要会展设施、专业批发市场、研发孵化机构落地我市。重点实施央企、外企和民企总部招商，针对北京准备外迁的千余家企业，精心确立目标，逐个实施攻关，坚决避免层次低、有污染的项目进入我市。积极推进高速、地铁、轻轨等重大交通设施对接，力促京台高速北京段、潮白河大桥等交通对接工程尽快开工。

　　四、不遗余力加快产业结构调整

　　瞄准绿色高端产业，突出实体经济项目，以项目带动、创新驱动为引擎，提升优势传统产业，培育壮大新兴产业，加快构建环保型产业体系。

　　（一）全力实施产业升级“四个五十”工程。结合省“三个一百”工程，在传统产业、战略性新兴产业、现代服务业和现代农业中，分别筛选50家经营规模大、创新能力强、发展前景好的企业，设立专项资金，开展专项帮扶，解决实际困难，支持做大做强。一是以改造提升50家传统产业企业为带动，加快传统产业转型升级，重点实施100项技改项目，拉动企业投资50亿元，年营业收入50亿元以上产业集群达到10个。二是以培育壮大50家战略性新兴产业企业为带动，下大力建设一批新一代信息技术、高端装备制造、生物医药、新材料、新能源等行业领军企业。三是以大力扶持50家现代服务业企业为带动，促进现代物流、研发设计、金融服务、云计算等生产性服务业发展，推进现代物流和金融后台园区建设，促进服务业与制造业融合发展。积极发展商贸、旅游、养老等生活性服务业，着力培育基于互联网的电子商务、文化创意、动漫游戏等新兴业态，大力改善消费环境，发挥消费对经济增长的促进作用。四是以做大做强50家现代农业企业为带动，大力发展蔬菜、粮油、畜牧、果品等优势农产品生产基地和加工业，不断延伸产业链条，形成集约化、科技化、规模化、品牌化的发展格局，提高我市优质农产品在首都市场的占有率，建设100个千万元以上农业产业化项目，力争农业产业化经营收入达到330亿元。

　　（二）力推项目建设上台阶。坚持以项目育产业、促调整、优结构，以更大的工作力度、更实的工作举措，全力推进项目建设。一是大力实施“十百千”工程。争列10个以上省级重点项目，继续保持全省领先，扎实推进100个市级重点项目和1000个县级重点项目建设，分级管理，明确责任，合力攻坚。力争每个县（市、区）至少建设5个10亿元项目、1个50亿元项目，全市建设3-5个百亿元项目。二是加快项目建设进度。围绕高端实体经济、重大产业聚集、京津冀战略合作，谋划储备一批重大项目，力促一批在谈重大项目尽快签约，推动中汽零产业基地、中海油研发基地、三河公务机场等一批已签约项目尽快开工，推进航天科技产业园、联通华北基地、精雕数控机床等一批在建项目加快建设、尽快投产达效。三是全力打好项目建设组合拳。强化领导推动，对重点项目实行市县党政领导分包，一个项目、一名领导、一套人马、一抓到底，实施月调度季拉练，集中观摩，强力推进。强化要素保障，围绕项目建设必需的土地、资金等问题，研究制定项目供地评价体系和金融支持体系，全面评估项目的技术水平、投资强度及贡献，对产出高、效益好的实体项目优先解决土地和融资困难。强化考核奖惩，把项目建设作为政绩考核的重要指标，排出位次，公开晾晒，落实奖惩。

　　（三）提升招商引资水平。深入开展招商引资集中行动，以环保型产业为基本方向，以京津地区为招商重点，有的放矢推进招商工作。一是强化招商意识，坚持各级干部一起上，各行各业一起上，央企外企民企一起上，建立企业库、项目库，制定招商路线图，掀起新一轮招商热潮。二是创新招商方式，统筹各园区专业力量，实施产业链招商和专业招商；以廊坊经洽会、“9·26”农交会、香港投洽会、厦门投洽会为平台，实施会展招商；以世界500强、国企百强、民企百强为重点，拉出单子、列出名录，实施点对点精准招商；充分发挥廊坊本地名企名人的作用，实施以商招商；以百家环保企业进廊坊、百家中关村企业进廊坊为重点，邀请万名客商来廊考察洽谈。三是增强招商实效，各级各部门主要领导要把招商引资工作放在心上、抓在手上，不仅亲自研究部署，还要亲自洽谈跑办，更要亲自协调督查，亲力亲为，一线指挥，千方百计争取一批立市立县项目落地。

　　（四）强化科技创新驱动能力。充分利用京津科技优势，着力建设四大创新平台。一是重大科技合作平台。支持燕郊和龙河高新技术产业开发区加快发展，积极推动清华大学等高校、科研机构重大科技成果孵化转化基地落户我市，加快河北航天遥感应用创新中心建设，推动北三县、固安与中关村合作建设科技成果转移基地，更好发挥清华研究院、科技谷、驻廊高校院所作用。引导金融信贷、风险投资进入农业科技创新领域，培育扶持农业科技成果托管中心和交易市场发展，进一步推动农业科技成果转化。二是创新孵化平台。加快廊坊市创新创业科技园、固安肽谷生命科学园、龙河高新区科技成果孵化园等平台建设，推动科技企业创业发展。三是企业研发平台。支持企业加大研发投入，加快推进企业技术中心、重点实验室等研发机构建设，提升大企业核心竞争力。加强对中小科技企业的政策及融资支持力度，培育一批科技“小巨人”。四是领军人才平台。完善促进创新、吸引人才的政策措施，为科技领军人才提供创业、居住、医疗、教育等优越条件，以优良的综合环境聚集高端人才。

　　五、攻坚克难推进环境治理和生态建设

　　把生态建设摆上事关全局的重要位置，以更加坚定的决心、更加过硬的措施，重拳治理污染，扎实推进全域性生态修复，全力打造平原森林城市，建设首都环境护城河。

　　（一）强力治理大气和水污染。深入开展大气污染治理集中行动，综合施策治理雾霾，在全省率先稳定退出全国重污染城市行列。一是严控污染源头。深化燃煤污染治理，全部完成工业企业燃煤锅炉脱硫除尘任务，强力淘汰市城区和各县（市）建成区燃煤茶浴炉和供暖小锅炉，市区城中村更换节能炉具和清洁型煤；加快国电廊坊热电厂建设，推动中电投热电厂尽快立项并开工建设。深化扬尘治理，提升城市环卫水平，高标准实施建筑施工“七个100%”，加强市区裸露土地的绿化硬化，减少二次扬尘。深化烟气治理，规模饭店全部安装油烟处理装置，全面禁止秸秆焚烧，推进秸秆综合利用，市主城区限时限区域禁放烟花爆竹、取缔露天烧烤。深化尾气治理，提前完成黄标车淘汰任务，全部完成加油站、油气库及油罐车的油气回收治理。深化工业污染治理，实施27项治污减排工程，对钢铁、水泥、电力、玻璃行业实施排污总量控制，上半年全部安装排污总量控制系统IC卡。二是强化应急机制。进一步完善重污染天气应急预案，加强应急组织机构建设，建立健全监测预警体系和专家会商研判机制，做到准确预警、科学应对。三是推进联防联治。实时发布各县（市、区）、廊坊开发区空气质量指数，加强与京津的协调联动。继续实施“利剑斩污”专项行动，强力执法，严格问责，坚决打击破坏环境的不法行为。推进“智慧环保”平台建设，全面构建环保网格化监管体系。四是开展水环境综合治理。研究制定市主城区水系治理总体方案并分期实施。启动水污染防治行动计划，重点开展污染源头治理，严厉打击暗管偷排和恶意倾倒废酸等违法行为，继续开展渗坑排查和综合整治。坚决控制地下水超采，加强对湿地的保护，支持香河等县（市）申报国家级湿地公园。进一步改善水环境质量，确保地表河流出境水质好于入境水质。

　　（二）坚决化解过剩产能。到2017年，我市要压减412万吨铁、170万吨钢、450万吨煤、156万标准重量箱平板玻璃，环首都县（市）所有水泥产能全部退出，任务十分艰巨。要制定过剩产能化解方案，强化保障措施，定计划、定指标，定时间表、定监督检查机制，层层分解任务，落实责任，全面完成今年化解过剩产能任务，淘汰水泥企业29家、产能1960万吨。加强钢铁行业整合重组，强化排放监管，确保达标排放。全面开展对岩棉、胶合板等重点行业的专项整治，对达不到环保要求的企业进行关停，对污染严重的设备实施拆除。到年底，所有企业都必须达到二级以上清洁生产标准，达不到标准的坚决淘汰。

　　（三）大力实施植树造林增绿工程。着眼建设平原森林城市，启动“两年攻坚战、造林一百万”专项行动。今年重点实施六大增绿工程，完成50万亩造林任务。重点建设京津廊交界生态森林景观带和高速公路、国省干道两侧高标准生态廊道，在永定河故道、文安洼地等区域成片建设生态林和经济林。在全市确定1000个村庄开展集中绿化，村庄绿化率达到45%以上。每个县（市、区）至少建设一个千亩以上骨干苗圃，全市新建苗圃1.5万亩。

　　六、奋发有为打造区域发展新引擎

　　紧紧围绕打造环京津新增长极战略突破口，着力激活全局关键点，挖掘经济增长点，点燃发展引爆点，构建经济发展新高地。

　　（一）推进临空经济区建设。按照高端化、国际化、高效化原则，坚持高标准规划、高起点发展，全力推进首都新机场和临空经济区建设。一是全力做好首都新机场建设涉及的征地拆迁、农民安置和配套迁改工作，加快推进土地组卷和回迁区规划建设，落实好农民搬迁扶持政策。二是积极配合国家和省编制临空经济区发展规划，争取更多体现廊坊利益诉求，适时修订我市临空经济区产业和空间规划，先期启动起步区详规编制，组建领导机构，搭建融资平台，充分做好前期工作。三是积极围绕临空经济区产业定位，超前储备一批好项目大项目，全力做好招商工作。

　　（二）推进北三县统筹发展。以“生态环境更好、商务成本更低、生活品质更优”为建设目标，加快北三县一体化发展步伐。一是规划统筹。按照交通是基础、生态是关键、产业是重点、城市是保障的规划思路，完成北三县统筹发展规划编制工作，确保北三县统筹发展在高水平规划指导下稳步实施。二是交通统筹。积极构建北三县高效互联的交通体系，重点打造以潮白河为中心轴，以“两横一纵”高速公路为主框架，以“三横两纵”干线公路为主动脉的路网系统，尽快打通跨县市的断头路，加强与京津的交通对接。三是环境统筹。以打造环京津最优的生态宜居环境为目标，加强植树造林和生态水系建设，实施北运河、潮白河综合治理和绿化美化，今年完成造林15万亩以上。四是产业统筹。以产业规划为引领，加快传统产业上层级、现代服务业上水平、战略性新兴产业上规模，提升产业集群发展质量，建设沿潮白河中央商务区、战略性新兴产业发展带和现代服务业示范区。

　　（三）建设廊南生态走廊和新型制造业基地。立足廊坊南部文安洼和霸州、大城部分地区的林业和湿地资源，谋划建设华北平原地区最大的生态走廊，形成京畿新的重要生态屏障，大力发展林产品生产加工和生态旅游，培育衍生新的富民兴县产业。立足南三县现有产业基础，以畅通交通为切入，主动接受空港、海港辐射，做大做强机械装备制造、家具生产、食品加工等传统产业，大力发展新型制造业，不断增强南部县（市）的整体发展水平。

　　（四）强化园区载体功能。坚持把园区作为支撑发展的基础平台，大力整合提升，促进项目和产业聚集。一是推进园区升级。以开发区和高新区为方向，整合提升各类园区。强化对后进园区的引导，加强基础设施建设，完善配套功能，破解规划档次低、承载功能弱、招商能力差的问题，支持各县集中力量打造1个精品园区，明确1-2个主导产业，迅速做大做强。二是创新园区管理模式。深入研究园区异地托管新思路，推动廊坊开发区一区多园建设。抢抓省支持环京津地区重点开发区和中外合作园区发展的有利时机，立足我市园区优势，积极推进环保产业园、管道产业园、电梯产业园等区中园发展，推动中外合作园区建设，力争年内新增1-2家中外合作园区。三是开展清理园区土地集中行动。对现有各类园区用地情况、开发强度、效益质量等进行综合评价，对所有项目供地逐宗调查清理，坚决依法收回闲置土地，提高开发强度和综合效益。

　　七、积极稳妥推进新型城镇化

　　立足廊坊良好基础和有利条件，坚持高起点规划、高水平建设、高效能管理，力争在城乡统筹发展上走在全省前列。

　　（一）有序推进农业转移人口市民化。按照全省统一部署，加快户籍制度改革，抓紧制定落户政策，有序放开廊坊市区落户限制，全面放开各县城、建制镇的落户限制。稳步推进城镇住房、就业、医疗、就学等基本公共服务常住人口全覆盖，逐步把进城落户的农民纳入城镇基本养老、医疗保险等社会保障体系，解决进城人员的后顾之忧。

　　（二）着力优化城镇布局。充分发挥规划的龙头作用，完善城镇规划，逐步由扩张性规划转向限定城市边界、优化空间结构规划，坚持城市组团发展方向。建立城乡统筹一体的规划体系，实现发展规划、城乡规划和土地利用规划“三规合一”。积极顺应京津冀协同发展趋势，推进环京津城镇群建设，不断优化城镇空间布局，中部以廊坊市区为核心，提升中心城市综合实力；北部以推进北三县统筹发展为重点，打造首都同城发展圈；南部以霸州为中心，建设天津都市功能协作区，构筑层级合理、各具特色、多点支撑、良性互动的城镇发展格局。注重城镇特色文化的保护、传承和挖掘，提升城镇内涵品位，让城镇有特色、有个性，历史文脉得以延续弘扬。

　　（三）提升中心城市承载力。强化城市功能，优化公共服务，彰显文化特色，提升城市品位。一是完善城市规划，加快城市总体规划报批，启动近期建设规划、新一轮控详规及重点专项规划编制工作。二是加快重点工程建设，以推进交通枢纽中心、苏宁广场等城建工程为重点，加快完善城市功能设施，以梦廊坊文化产业园、尖塔改造等工程为重点，加快推进生态文化艺术新区建设。三是加强城市精细化管理，着眼促进信息化与城镇化互动发展，整合各类数字化平台，加快建设智慧城市，拓展数字城管功能，推进地理信息共享服务平台在规划、交警、综合执法等多部门联合应用，不断提升城市管理精细化水平。四是全面改善城市环境，深入开展城区面貌整治集中行动，以治脏、增绿、有序为重点，打造整洁宜居的生态环境。

　　（四）加快县域经济发展和县城建设。以增强县域综合承载能力和提高宜居水平为目标，全面落实支持县域经济发展和县城建设的政策措施。实施新一轮县城规划，全面完成各县（市）城乡总体规划编制，加紧完成控详规和各专项规划编制。在县城重点区域、重点节点，集中力量打造一批精品工程、地标性建筑和特色街区；完善集中供热、污水管网、垃圾处理等配套设施，集中供热普及率达到80%以上；加快建设图书馆、博物馆、规划馆等公共设施，谋划建设城市综合体项目。以县城建设带动县域经济发展，坚持产城互动融合，着力培育一批特色主导产业、精品园区和龙头企业，带动县域经济发展。

　　（五）积极推进新农村建设。以城乡一体化发展为目标，加快发展农村经济，改善农村面貌，促进新农村建设迈上新台阶。一是加快现代农业园区建设。以现代农业为主攻方向，完善组织服务、金融助农、科技支撑三大体系建设，构建环绕北京的现代农业园区体系。推进三河国家级农业园区和安次、大厂省级农业园区建设，支持借鉴境外先进农业技术的中外、冀台合作农业园区发展，实施10个农业综合开发项目，力争年内建成5个年销售收入超亿元的现代农业示范园区。推进环首都经济圈绿色蔬菜基地建设，抓好7个蔬菜产业示范县、40个省级蔬菜示范区建设，发展10个千亩以上现代蔬菜产业园，农业产业化经营率达到67%。二是改善农村生产生活条件。加大农业基础设施投入，年内新建、改造公路120公里，大力发展高标准设施农业、高效节水农业，三年发展高效节水设施农业50万亩，全市农业机械化水平达到69%。三是深入实施农村面貌改造提升行动。进一步巩固和深化“四清四化”成果，建立完善农村环境卫生管护机制。以建设428个省级重点村和20个精品村为抓手，全面推进环境治理、村庄绿化等六大工程，高标准实施道路硬化、垃圾处理、饮水安全等15件实事，打造廊坊新农村的升级版。

　　八、持续努力保障和改善民生

　　民之所望，施政所向。我们一定把改善民生作为根本，坚持优先保障民生投入、优先安排民生项目、优先解决民生问题，让发展成果更多更公平惠及全市人民。

　　（一）努力提高城乡居民收入。增加城乡居民工资性收入，完善最低工资和工资支付保障制度，推行企业工资集体协商制度，将工资集体协商建制率和职工工资增长率纳入各县（市、区）及有关部门的考核范围。进一步提高企业退休人员基本养老金水平。大力发展非农产业，增加财政对“三农”的转移支付，进一步增加农民收入。

　　（二）千方百计扩大就业。深入实施就业优先政策，完善公共就业服务体系，年内城镇新增就业4.9万人，城镇登记失业率控制在4%以内。着力抓好高校毕业生就业工作，落实大学生创业扶持政策，应届高校毕业生就业率达到87.5%以上。推进农村劳动力就近就业和返乡创业，完成农民培训20万人次，新增农村劳动力转移就业2.8万人。开展就业困难群体就业援助，确保城镇零就业家庭动态归零。

　　（三）健全完善社会保障体系。实施社保扩面提升工程，健全完善城乡居民基本养老保险制度，加快养老服务体系建设和养老产业发展，将养老服务补贴制度扩展到各县（市），扩大城市社区养老服务中心和农村互助幸福院覆盖范围，鼓励小微企业、个体工商户参加企业基本养老保险，多元增加养老投入。新农合筹资标准提高到390元，农民住院补偿最高支付限额提高到10万元，新农合参合率保持在95%以上。城镇低保保障标准由月人均422元提高到532元，农村低保标准由232元提高到292元。五保户集中供养标准由年人均3500元提高到5750元，分散供养标准由3384元提高到5000元。加大对低保对象等困难群众医疗救助力度，低保对象医疗报销最高比例由60%提高到80%，医疗救助封顶线由2万元提高到3万元，医疗救助“一站式”即时结算服务体系实现全覆盖。落实惠民殡葬政策，加快市殡仪馆规划建设。

　　（四）加快改善就学就医条件。办好群众满意的教育，加快市城区教育扩容提质，廊坊一中迁建工程开工建设，改扩建3所小学，新建3所中小学和5所幼儿园。逐步缩小城乡和校际差距，县域投资10.2亿元实施63个学校建设项目，全市建设标准化学校140所，力争40%的中小学和幼儿园实现标准化。大力加强师资队伍建设，全面提升教育教学水平，立足特色加快发展职业教育，创造条件积极支持域内省属和市属高校做大做强。进一步完善城乡医疗卫生服务体系，开工建设市中心医院，加快卫生信息平台和卫生应急指挥平台建设，市区建设一批社区卫生服务中心，强化县域公立试点医院医疗卫生服务和紧急救援职能，努力实现大病不出县。

　　（五）优化群众居住环境。加大住房保障和旧城改造力度，建设保障性住房1.7万套，完成2475户农村危房改造，主城区实施管道小区、华阳里等20个旧小区、棚户区、无物业小区改造。进一步提高居民生活便利度，新开通5条公交线路，推进市区停车场规划编制和工程建设，实施一批小游园、小停车场、小街巷改造以及便民市场等项目建设，让群众生活更方便、更舒心。进一步提高居民生活环境质量，实施自然公园和文化公园改造提升工程、城市出口和迎宾线绿化提升工程、永定河绿廊工程以及广阳道、爱民道等重点干道绿化亮化工程，大力营造宜人的城市景观。进一步强化城乡供水保障，南水北调保沧干渠达到通水条件，市县8座地表水厂9月前建成投用，积极谋划南水北调北三县供水工程。启动市区新水源地迁建工程，全面完成农村水厂管网建设，严格保障饮水安全，让群众喝上安全水、放心水。

　　（六）进一步丰富群众文化生活。积极创建国家公共文化服务体系示范区，着力提升市县乡村四级公共文化基础设施，加快市群艺馆、美术馆建设，组织好幸福廊坊文化艺术节及城市广场文化活动，扶持基层文化社团发展，让群众共享文化发展成果。广泛开展群众性体育健身活动，着力推进市体育公园、市区绿道健身工程建设，健身设施覆盖全市75%社区和50%村街，积极备战省第十四届运动会和第八届残运会。

　　（七）全力维护社会和谐稳定。切实强化安全生产和食品药品安全，坚决遏制重特大事故发生，确保群众“舌尖”上的安全。切实强化矛盾隐患排查化解，开展信访积案化解集中行动，90%以上积案实现案结事了、息诉息访。切实强化平安廊坊建设，实施“四个治理”，搞好社会治安防控，积极开展身边小案集中破解行动，有效净化社会治安环境。切实加强信用体系建设，以政务诚信引领社会诚信，加大重点领域信用建设力度，提高市场主体失信成本，筑牢信用之城。切实加强国防后备力量建设和拥军工作，搞好人口综合服务和管理，做好工会、物价、审计、人防、气象、援疆、妇女儿童、民族宗教、防灾减灾等工作，促进各项事业共同发展。

　　各位代表！做好今年的政府工作，要以深入开展以为民务实清廉为主题的党的群众路线教育实践活动为契机，认真贯彻落实中央“八项规定”，坚决反对和克服“四风”，以改革创新精神加强政府自身建设，全力推进各项工作落实。我们一定坚持勤政为民，着力建设服务政府。政府所有工作都必须以服务群众为根本、以群众满意为评判标准，想群众之所想，急群众之所急，多做雪中送炭、排忧解难的好事实事，用勤勉的敬业精神、扎实的工作作风、实在的发展成果取信于民。我们一定坚持求真务实，着力建设实干政府。力戒空谈，崇尚实干，大力精简会议和文件，腾出时间精力，多蹲点调研，多下访解难，多上门服务，扑下身子，真抓实干，坚持多做少说、只做不说，努力营造让群众和投资者放心、安心、舒心的生产生活环境。我们一定坚持依法行政，着力建设法治政府。落实重大行政决策程序规定，完善行政决策风险评估机制，着力提高政府决策科学化、民主化、法治化水平。更加自觉地接受人大法律监督、工作监督和政协民主监督，强化舆论和社会监督，使权力运行在法治的轨道上。我们一定坚持廉洁从政，着力建设廉洁政府。深入推进政府系统惩治和预防腐败体系建设，认真落实党风廉政建设责任制，坚持“一岗双责”。全面压缩一般性支出，各级政府会议费压减10%，培训费和“三公”经费压减5%以上，把更多财力用于保障和改善民生，在政府自觉过“紧日子”中，让群众过上“好日子”。

　　各位代表，我们的政府是人民的政府，政府的权力来自人民的信任，政府的职责是全心全意为人民服务。我们将把人民的向往作为政府工作的目标和方向，受人民所托，忠人民之事，办人民所需，尽职尽责，尽心尽力，为人民谋福祉。

　　各位代表，站在新的起点上，我们肩负的任务艰巨、使命光荣。让我们紧密团结在以习近平同志为总书记的党中央周围，在省委、省政府和市委坚强领导下，锐意进取，敢于担当，干在实处，走在前列，全力夺取2014年各项工作的新胜利，谱写廊坊绿色发展、高端发展、率先发展、和谐发展的新篇章！
